# Effects of Different Types of COVID-19 Vaccines on Menstrual Cycles of Females of Reproductive Age Group (15-49): A Multinational Cross-Sectional Study

**DOI:** 10.7759/cureus.39640

**Published:** 2023-05-29

**Authors:** Nuha N Filfilan, Suhaib Bukhari, Maryam Rizwan, Nirmeen M Bukhari, Nisreen K Aref, Farzana R Arain, Ibrahim K Alabbadi

**Affiliations:** 1 Public Health and Preventive Medicine, Taif University, Taif, SAU; 2 Obstetrics and Gynaecology, University of Jeddah, Jeddah, SAU; 3 Obstetrics and Gynaecology, Shaheed Zulfiqar Ali Bhutto Medical University, Islamabad, PAK; 4 Obstetrics and Gynaecology, King Abdulaziz University Faculty of Medicine, Jeddah, SAU; 5 Obstetrics and Gynaecology, Taif University, Taif, SAU; 6 Health Services and Hospital Administration, Taif University, Taif, SAU

**Keywords:** types of covid-19 vaccines, reproductive age, menstrual irregularities, covid-19 vaccines, vaccine side-effects

## Abstract

Background

Globally, there are more than 474 million cases and around 6 million deaths due to COVID-19. The case fatality rate was 0.5-2.8% while for 80-89 years old, it was 3.7-14.8%. Given the seriousness of this infection, prevention becomes critical. Hence, the introduction of vaccines led to a significant reduction (> 75% protection) in COVID-19 cases. On the other hand, patients seeking help for serious pulmonary, cardiovascular, neurological, and gynecological complaints have also been recorded. Clinical studies on the effects of vaccination focused mostly on life-or-death results rather than reproductive outcomes such as menstruation, fertility, or even pregnancy outcomes. This survey was conducted to get more evidence on the association between menstrual cycle irregularities and some globally most prevalent COVID-19 vaccines.

Methods

An online cross-sectional survey was conducted by a team from Taif University, Kingdom of Saudi Arabia, from January to June 2022 on females within the reproductive age group (15-49 years) using a semi-structured questionnaire. Data were analyzed using SPSS Statistics version 22.0 and presented as frequency and percentage. The chi-square test was applied for the association and a p-value of <0.05 was considered significant.

Results

A total of 2381 responses were included. The mean age of respondents was 25±7.7 years. Around 1604 (67%) participants observed post-vaccination menstrual changes, and the findings were significant (p< 0.001). A strong association (p=.008) was found between the type of vaccine and changes in the menstrual cycle in participants (AstraZeneca 11 (36%)) after one dose. A strong association (p=.004) was also seen between the type of vaccine (Pfizer 543 (83%)) and menstrual changes after the booster dose. Cycles became irregular 180 (36%) or prolonged 144 (29%) in females inoculated with Pfizer after two doses of vaccination (p=0.012).

Conclusion

Post-vaccination menstrual irregularities were reported by females of reproductive age, especially the new vaccines. Prospective studies for similar insights are needed. Finding the co-occurring impacts of vaccination and COVID-19 infections in the wake of the emerging new long-haul COVID-19 phenomena is crucial for reproductive health.

## Introduction

COVID-19, caused by the agent SARS-CoV-2, is an infection of the respiratory tract that spreads through droplets and has high transmissibility. Patients with COVID-19 infection present with a wide range of clinical symptoms. The majority (80%) of patients usually experience mild to moderate respiratory symptoms, while in 15% of cases, symptoms are severe, and 5% end up with critical life-threatening multiple organ dysfunctions. As of March 24, 2022, the WHO has confirmed more than 474 million cases globally, with around 6 million deaths due to COVID-19. Furthermore, the global case fatality rate ranges from 0.3% to 2.8%, which decreased from 1.7% to 39.0% in 2020 [1,2].

Given the seriousness of this disease, treatment becomes critical, and there has been significant pressure on the healthcare system. Proper therapeutic strategies are still not available, and the few therapies with confirmed benefits are very expensive and not readily available. In an effort to contain the infection, the basic approach adopted in most parts of the world was the use of non-pharmaceutical interventions (NPIs), such as wearing masks, observing hand hygiene, practicing cough and respiratory etiquette, social distancing (restricting movement, avoiding crowds, working from home), closure of academic/educational institutions, contact tracing, home isolation, and lockdowns [3]. Although the implementation of NPIs significantly contributed to flattening the case curve, the role of antiviral drugs and prophylactic vaccines cannot be ignored. Today, the capacity of medical technology to test already available and new antiviral drugs and develop safe, efficacious, and readily available vaccines at a reasonable price is more important than ever. Out of the two methods, vaccines have proven to be the more reliable and cost-effective public health intervention, primarily to contain and mitigate not only the virus itself but also the economy and social fabric of society [4,5].

The introduction of vaccines has led to a significant reduction in clinically significant coronavirus disease cases, as well as the number of asymptomatic infections and the associated infectivity [6]. WHO has so far approved several vaccines that are being used across the globe. Most vaccines are designed for two-dose schedules, while a few are single doses. To stimulate the immune system to produce an appropriate response to fight the disease, vaccines are developed using different technologies. Many vaccines like Sinopharm, Sinovac, and CanSino use a weakened or inactivated virus to trigger the immune cell response, while relatively new Pfizer and Moderna vaccines make use of mRNA to signal the immune cells to produce the "spike protein," a protein present on the surface of SARS-CoV-2 and an antigen against which antibodies are produced. AstraZeneca and Sputnik use a modified virus (known as adenovirus or vector virus) that carries the genetic material to ultimately produce the "spike protein" and trigger an immune response [6].

The efficacy of a vaccine largely depends on its role in impeding the transmission of the pathogen and limiting the progression of the disease [7]. Evidence from phase 4 clinical trial data has shown that so far Pfizer and Moderna are the most efficacious vaccines (95%), followed by Sputnik (91.6%) and CanSino (92%). The efficacy of Sinopharm was calculated to be 85.7%, while for AstraZeneca, it was 85.6% [8]. Overall, receiving two doses of COVID-19 vaccines offers more than 75% protection against all symptomatic diseases, while a single dose provides 79% protection from severe illness and hospitalization [9]. Despite these satisfactory figures, breakthrough infections and adverse events after vaccination are being reported from different parts of the world [10].

Due to the diversity and unpredictability of COVID-19 symptoms, different individuals require different types of post-COVID care. Some adverse effects following immunization are common and typically not alarming in nature. In addition to fatigue, chills, joint pain, and nausea, several participants in COVID-19 vaccine trials experienced tenderness, pain, warmth, itching, or bruising at the injection site [10]. On the other hand, studies have reported patients seeking help for serious pulmonary, cardiovascular, neurological, and gynecological complaints [11-14].

One of the adverse effects among women of childbearing age is menstrual cycle disturbances, which need to be identified and explored [1]. It is important that all women, regardless of whether they are expecting or not, receive the COVID-19 vaccine. It reduces the risk of contracting the virus and helps protect both mother and fetus during pregnancy. However, some people are reluctant to get vaccinated due to concerns about the vaccine's potential effects on menstruation [3]. Reports of menstrual disturbances after COVID-19 vaccination have been documented from around the world [3]. Several women have claimed on various social media platforms that their menstrual cycles changed after vaccination, becoming heavier, starting sooner, or being more painful. However, clinical studies conducted to examine the effects of vaccination primarily focused on life-or-death outcomes rather than reproductive outcomes such as menstruation, fertility, or pregnancy outcomes [15].

Menstrual alterations occurring after immunization remain a mystery. The endocrine system, which is involved in reproduction, and the immune system share similarities that may hold the answer [15-17]. In fact, immunological activity in response to various stimuli, including viral infection, can impact the menstrual cycle. Disruption of regular monthly cycles in females after COVID-19 infections and vaccination has also been reported in several studies [11,16]. Reports from different sources have shown that females who have been inoculated with the COVID-19 vaccine have experienced various menstrual changes, including menorrhagia, oligomenorrhea, and dysmenorrhea [16-18]. It has also been mentioned that those who received the vaccine twice in one menstrual cycle reported that their periods came a few days later [19]. However, most of these reported alterations were brief, as their periods returned to normal after a few cycles [18-19].

According to experts, it is important to conduct population-based prospective studies and randomized clinical trials to investigate the impact of COVID-19 vaccination on menstrual cycles [18]. Awareness of these events is necessary to foster confidence in vaccination. Failing to thoroughly investigate reports of menstrual changes after vaccination is likely to fuel fears of adverse effects. If a link between vaccination and menstrual changes is confirmed, this information will allow people to plan for potentially altered cycles. Clear and reliable information is also crucial, especially for females who rely on their ability to predict their monthly periods for pregnancy planning or who prefer natural methods of contraception [20].

This online survey was conducted to gather more evidence on the association between menstrual cycle irregularities and COVID-19 vaccination. Additionally, we planned to further analyze the data to assess the impact of common variables on the link between COVID-19 vaccines and irregularities in menstrual cycles.

## Materials and methods

Study type, settings, and duration

An online cross-sectional survey was conducted by a team from Taif Medical College, University of Taif, Kingdom of Saudi Arabia, from January to June 2022 on females of the reproductive age group (15-49 years). The survey was conducted in a multinational setting, encompassing participants from various countries. Females from the Kingdom of Saudi Arabia, Pakistan, Bahrain, United Arab Emirates, Kuwait, Iraq, Egypt, Dubai, Sri Lanka, Jordan, Algeria, Australia, Serbia, and Tunisia took part in the survey. This diverse group of countries represents a wide range of geographic locations, cultural backgrounds, and socio-economic contexts.

Sampling technique

A non-probability convenient sampling technique was used. The sample size of 2,426 was calculated by taking reference to post-vaccination (COVID-19) menstrual abnormality in one-fifth of the study participants [21], at a 95% confidence level, 2% margin of error, 1.5 design effect, 5% non-response rate, and 5% margin of error.

Inclusion criteria

Females of the reproductive age group, i.e., 15-49 years, with a normal menstrual cycle (25-35 days) before vaccination, who had received at least one dose of COVID-19 vaccine at least one month before participating in this survey, and had access to the Internet and social media platforms on their mobile phones. Furthermore, females with a minimum of three cycles after delivery/pregnancy and with known geographical locations were also included.

Exclusion criteria

Pregnant females, females suffering from amenorrhea, females using oral contraceptives for more than five months, and females with polycystic ovary syndrome were excluded.

Tool development

The questionnaire was developed by the core team along with subject matter experts. The questionnaire comprised 42 variables, including age, age of menarche, number of doses received along with the name of the vaccination, duration between the different doses of COVID-19 vaccination received, and pre-vaccination information about the menstrual cycle in terms of regularity, length, duration, and volume. Females were also asked to provide information about post-vaccination (even after the first dose) changes in the length of the cycle, the flow of menstrual bleeding, dysmenorrhea, and changes in its intensity and intermenstrual bleeding. The content and construct validity of the developed tool were assessed by sharing the questionnaire with six to eight experts in the field. The experts marked each question in terms of relevance and clarity. Variables that scored more than 80% for content and construct validity were included, while questions receiving less than 80% score were revised and finalized for the survey.

Ethical consideration

Ethical approval was obtained from Taif University's Scientific Research Ethics Committee (approval number 43-050). As this was an online survey, a consent form was included at the beginning of the questionnaire. It clearly stated the study objectives, emphasized voluntary participation and the absence of monetary benefits, and assured participants that their personal information would be kept confidential and used only for research purposes. Since all study participants were literate, only those who understood and agreed to the ethical statement proceeded further with the full-length survey. The questionnaire was also translated into Urdu and Arabic, and participants were given the option to choose their preferred language for filling out the questionnaire.

Data collection

The validated questionnaire was filled out using Google Forms, and the link was posted on social media platforms, primarily through WhatsApp, to females of reproductive age groups. Volunteer data collectors helped distribute the questionnaire further to obtain more responses.

Data analysis

Data were analyzed using SPSS Statistics for Windows version 22.0 (IBM Corp. Released 2013. IBM SPSS Statistics for Windows, Version 22.0. Armonk, NY: IBM Corp.). Categorical data were presented as frequency and percentage. The chi-square test was applied to observe the association between variables and a p-value of <0.05.

## Results

Out of 2,426 participants, those who filled in less than 60% of the information on the questionnaire were excluded, resulting in a total of 2,381 responses included in the study. The majority of the participants, 1,679 (70%), were from the Kingdom of Saudi Arabia, 563 (24%) were Pakistani, 56 (2%) were from Bahrain, 25 (1%) were from the United Arab Emirates, 21 (1%) were Kuwaiti nationals, 16 (1%) each were from Iraq and Egypt, five (0.2%) each were from Dubai and Sri Lanka, four (0.1%) each were from Jordan and Algeria, three (0.1%) were from Australia, and two (0.1%) were from Serbia and Tunisia. The majority of participants, 2,164 (91%), resided in urban areas. The mean age of our study respondents was 25±7.7 years, and the majority, 1,060 (45%), were in the age group ranging from 21 to 30 years, followed by 866 (36%) in the range of 15-20%. The age at menarche reported by 2,176 (91%) females ranged between 11 and 15 years. The majority, 1,785 (75%), of the females were single, while 531 (22%) were married. Most, 1,475 (62%), study participants had received secondary education, followed by a bachelor's degree, 527 (22%), while 207 (9%) had postgraduate/master's degrees. The menstrual cycle of the females before they received any vaccination was regular. Among them, 2,009 (84%) participants had periods that lasted 3-10 days, while 1,667 (70%) reported not having any discharge between the cycles. Additionally, 1,790 (75%) reported mood changes, and 2,067 (87%) had dysmenorrhea. According to the numeric rating scale, out of the 2,067 (87%) who experienced pain, 358 (15%) had mild pain, 910 (38%) had moderate pain, and 899 (38%) had severe pain. Among our study population of 360 (11%), 206 (58%) females suffered from anemia, 22 (6%) participants had spine, brain, and neurological problems, and 18 (5%) reported having blood pressure issues (Table 1).

**Table 1:** Socio-demographic and clinical characteristics of the study sample (N=2,381)

Parameters		n	%
Nationality	Kingdom of Saudi Arabia	1659	69.6
	Pakistan	563	23.6
	Bahrain	56	2.3
	Others	103	4.3
Social background	Urban	2164	90.9
	Rural	217	9.1
Age	15 to 20	859	36.4
	21 to 30	1060	44.5
	31 to 40	288	12.1
	41 to 49	167	7
Age at menarche	6-10	176	7.4
	11 to 15	2176	91.4
	16 ≥	29	1.2
	< High school/technical education	208	8.7
	Secondary/high school	1475	61.9
	Bachelors	527	22.1
	Post-graduate/master's degree	207	8.7
Marital status	Single	1785	75.0
	Married	531	22.3
	Widow	34	1.5
	Divorced/separated	31	1.3
Any medical problem	Yes	360	15.1
	No	2021	84.9
	Anemia	206	8.7
	Spine, brain, and neurological problems	22	0.9
	High and low blood pressure	18	0.8
	Allergies, psoriasis, eczema	15	0.6
	Depression and anxiety	15	0.6
	Diabetes mellitus	14	0.6
	Kidney issues, eye issues, others	14	0.6
	Gastrointestinal tract problems	12	0.5
	Heart problem	11	0.5
	Arthritis	8	0.3
	Asthma and breathing issues	8	0.3
	Vitamin deficiency	6	0.3
	Blood disorders: thalassemia, DIT	6	0.3
Vaccination status	One dose	50	2.1
	Two doses	1362	57.2
	Three doses	969	40.7

Regarding the vaccination status, 1,362 (57%) study participants had received 02 doses, while 969 (41%) received the booster dose as well, and 50 (2%) received only one shot of the vaccination. The different types of vaccines that the participants received included Pfizer-BioNTech, received by 1512 (64%) individuals, Sinovac by 389 (16%), AstraZeneca/Oxford by 199 (8%), Sinopharm by 193 (8%), Moderna by 81 (3%), and Sputnik by 7 (1%). The average duration between the first and second dose was 49±56 days. The average time since the participants were last vaccinated was 188±350 days.

Around 1,604 (67%) study participants reported changes in their menstrual cycle after receiving COVID-19 vaccinations. Approximately 357 (22%) study participants reported changes after the first dose, 876 (55%) after the second dose, and 13 (1%) females after the booster, and these findings were statistically significant (p<0.001) (Table 2).

**Table 2:** Post-vaccine changes in menstruation (N=2,381)

No of doses	Change in the menstrual cycle				
	No n(%)	Yes n(%)	Total n(%)	p-value	
No change reported	462 (59.5)	83 (5.2)	545 (22.9)	.001	
After one dose	3 (0.4)	357 (22.3)	360 (15.1)		
After two doses	24 (3.1)	876 (54.6)	900 (37.8)		
After booster	0 (0.0)	13 (0.8)	13 (0.3)		
Could not recall	288 (37.1)	275 (17.1)	563 (23.6)		
Total	777 (100.0)	1604 (100.0)	2381 (100.0)		

A significant association (p=0.008) was found between the type of vaccine and the reported changes in the menstrual cycle by the participants who received one dose of the vaccine. The results showed that changes were mostly reported by females who received AstraZeneca, with 11 (36%) participants, followed by Pfizer with 9 (29%) and Sinovac with 7 (23%). A strong association (p=0.004) was also observed between the type of vaccine and menstrual changes in participants who received booster doses, with Pfizer being at the top of the list with 543 (83%) participants (Table 3).

**Table 3:** Vaccination status, vaccine type, and changes in menstrual cycle (N=2,381)

Vaccine status	Vaccine type	Change in menstrual cycle			p-value
		No n(%)	Yes n(%)	Total n(%)	
One dose (n=50)	Sinovac	2 (10.5)	7 (22.6)	9 (18)	0.008
	Pfizer	10 52.6	9 (29)	19 (38)	
	AstraZeneca	1 (5.3)	11 (35.5)	12 (24)	
	Sinopharm	1 (5.3)	3 (9.7)	4 (8.0)	
	Moderna	5 (26.3)	1 (3.2)	6 (12)	
Two doses (n=1362)	Sinovac	109 (24.3)	229 (24.9)	338 (24.7)	0.076
	Pfizer	215 (47.9)	496 (53.9)	711 (51.9)	
	Astrazenica	64 (14.3)	115 (12.5)	179 (13.1)	
	Sinopharm	26 (5.8)	33 (3.6)	59 (4.3)	
	Moderna	28 (6.2)	40 (4.3)	68 (5)	
	Sputnik	0 (0.0)	7 (.8)	14 (1.0)	
Booster (n=969)	Sinovac	10 (3.2)	32 (4.9)	42 (4.3)	0.004
	Pfizer	239 (75.6)	543 (83.2)	782 (80.7)	
	AstraZeneca	5 (1.6)	3 (.5)	8 (.8)	
	Sinopharm	59 18.7)	71 (10.9)	130 (13.4)	
	Moderna	3 (.9)	4 (.6)	7 (.7)	
Total		777	1604	2381	

Different abnormalities in menstruation were observed among the study population following the COVID-19 vaccination. The majority, 1,685 (56%), of the changes were related to the duration of the menstrual cycles, which became either irregular (unscheduled bleeding) in 596 (35%) cases, prolonged (longer than 35 days) in 476 (28%) cases, or short in 311 (18%) cases.

The data were stratified based on the number of doses received, and the different changes and abnormalities in the menstrual cycle were analyzed according to the type of vaccines. Changes in menstrual cycles were noticed for all types of vaccines. However, in the majority of cases, 180 (36%) of the study participants, the cycle became irregular, and in 144 (29%) cases, it became prolonged (longer than 35 days) after receiving two doses of the Pfizer vaccine (mRNA design/technology-based) (p=0.012). Abnormalities were also experienced in the quality of menstrual blood, such as clot formation in 989 (42%) cases, and the quantity of menstrual flow, with heavy flow reported in 745 (31%) cases. Another change highlighted by the participants was an increase in the intensity of pain, shifting from moderate in the majority, 910 (38%), to severe in 1,159 (49%) cases after immunization. A strong association was found between severe dysmenorrhea and post-vaccine menstrual changes in 656 (47%) cases (p<0.001) (Table 4).

**Table 4:** Vaccination status, vaccine type, and types of changes in menstrual cycle (N=2,381)

Vaccine status	Vaccine type	Types of changes in menstrual cycle										p-value
		No change n(%)	Heavy bleeding n(%)	Less bleeding n(%)	Irregular cycle n(%)	Short cycle n(%)	Prolong cycle n(%)	Pain during cycle n(%)	Bleeding between cycle n(%)	Other n(%)	Total n(%)	
One dose (50)	Sinovac (9)	6 (40.0)	-	-	3 (20.0)	2 (13.3)	2 (13.3)	2 (13.3)	-	-	15 (100)	0.654
	Pfizer (19)	11 (40.7)	1 (3.7)	-	5 (18.5)	4 (14.8)	4 (14.8)	2 (7.4)	-	-	27 (100)	
	AstraZeneca (12)	3 (20.0)	-	-	4 (26.7)	2 (13.3)	-	4 (26.7)	-	-	13 (100)	
	Sinopharm (4)	1 (20.0)	-	-	3 (60.0)	-	-	1 (20.0)	-	-	5 (100)	
	Moderna (6)	5 (71.4)	-	-	1 (14.3)	-	-	1 (14.3)	-	-	7 (100)	
Total		26 (37.7)	1 (14.3)	-	16 (23.2)	8 (11.6)	8 (11.6)	10 (14.5)	-	-	69 (100)	
Two doses (1362)	Sinovac (338)	112 (32.3)	20 (5.8)	8 (2.3)	86 (24.8)	41 (11.8)	70 (20.2)	10 (2.9)	-	-	347 (100)	0.012
	Pfizer (711)	223 (30.8)	38 (5.2)	9 (1.2)	180 (24.9)	106 (14.6)	144 (19.9)	15 (2.1)	9 (1.2)	-	724 (100)	
	AstraZeneca (179)	63 (33.3)	11 (5.8)	2 (1.1)	39 (20.6)	28 (14.8)	39 (20.6)	6 (3.2)	1 (.5)	-	189 (100)	
	Sinopharm (59)	26 (38.8)	3 (4.5)	3 (4.5)	13 (19.4)	9 (13.4)	8 (11.9)	5 (7.5)	-	-	67 (100)	
	Moderna (68)	29 (38.2)	3 (3.9)	2 (2.6)	22 (28.9)	4 (5.3)	14 (18.4)	2 (2.6)	-	-	76 (100)	
	Sputnik (7)	-	3 (20.0)	-	6 (40.0)	1 (6.7)	2 (13.3)	3 (20.0)	-	-	15 (100)	
Total		453 (31.9)	78 (5.5)	24 (1.7)	346 (24.4)	189 (13.3)	277 (19.5)	4 (12.9)	10 (.7)	-	1418 (100)	
Booster (969)	Sinovac (42)	10 (27.8)	2 (5.6)	2 (5.6)	11 (30.6)	3 (8.3)	7 (19.4)	-	1 (2.8)	-	36 (100)	0.089
	Pfizer (782)	239 (30.4)	48 (6.1)	16 (2.0)	194 (24.7)	97 (12.3)	158 (20.1)	21 (2.7)	4 (.5)	9 (1.1)	786 (100)	
	AstraZeneca (8)	5 (33.3)	1 (6.7)	1 (6.7)	2 (13.3)	1 (6.7)	2 (13.3)	3 (20.0)	-	-	15 (100)	
	Sinopharm (130)	61 (41.2)	16 (10.8)	5 (3.4)	26 (17.6)	12 (8.1)	23 (15.5)	4 (2.7)	1 (.7)	-	148 (100)	
	Moderna (7)	3 (30.0)	2 (20.0)	1 (10.0)	1 (10.0)	1 (10.0)	1 (10.0)	-	1 (10.0)	-	10 (100)	
Total		318 (32.0)	69 (6.9)	25 (2.5)	234 (23.5)	114 (11.5)	191 (19.2)	28 (2.8)	7 (.7)	9 (.9)	995 (100)	

The majority of the study population, with ages ranging from 21 to 30 years, experienced post-vaccine menstrual changes, and the results were highly significant (p=0.05). Similarly, another statistically significant finding (p=0.001) was the impact of the level of education on the menstrual cycle of COVID-19-vaccinated females. Post-vaccination changes in the menstrual periods were experienced by 996 (71%) females who had completed their secondary school education.

## Discussion

The objective of this study was to estimate the effect of COVID-19 vaccination on the menstrual cycle of women of reproductive age (15-49 years).

Our findings suggest that post-COVID-19 vaccination changes in the menstrual cycle were observed in 1,604 (67%) study participants. Among these females, menstrual abnormalities were reported by 876 (55%) after the second dose, followed by 357 (22%) after the first dose and 13 (1%) after the third dose. Although data suggest that the incidence of menstrual abnormalities was already relatively high in different parts of the world before the COVID-19 pandemic and vaccination initiation, a recent study published in 2021 from Japan reported an incidence of menstrual abnormalities of 34% among women of reproductive age in 2017, when there was no pandemic [22]. The literature shows that the incidence of menstrual changes after COVID-19 vaccination varies from 21% to 68%, and our findings are consistent with published data. Biologically, there is a plausible link between the activation of the menstrual cycle by various stimuli, including vaccination [15,23]. This link is supported by several studies, including a recent prospective study conducted in the US, which found a significant association between menstrual cycle abnormalities and COVID-19 vaccination [24]. Another hypothesis is that the strong immune reaction and stress after COVID-19 vaccination may affect the hypothalamic-pituitary-ovarian axis, resulting in menstrual abnormalities [24].

One important finding of our study is that the type of vaccine significantly influences the incidence of post-vaccination menstrual abnormalities. Similar findings have been reported by Kareem et al. [25], although Lagana et al. reported no menstrual complications with different types of vaccines among their study participants [26].

Our study shows that monthly cycles were altered with all vaccines, but the majority of women received either AstraZeneca (adenovirus vectored design/technology-based) or Pfizer (mRNA design/technology-based) vaccine. Since relatively higher rates of irregularities in monthly cycles have been reported in other studies following injections of these vaccines, further research is needed to investigate the extent of these abnormalities and their potential consequences [27-29].

In the current study, in addition to irregularities, another significant abnormality in menstruation after COVID-19 vaccination was an increase in the length of the menstrual cycle in 469 (34%) study participants. This is consistent with the findings of a large prospective cohort study conducted in the US, which showed a non-persistent increase in the average length of menstrual cycles among COVID-19-vaccinated females [30].

A strong association was found between age and post-vaccination changes in the menstrual cycle in this study. A significant number of women in the 21-30 years age group reported these changes. Our results are consistent with Alghamdi et al., who reported menstrual changes in females of the same age group after receiving the COVID-19 vaccine [30]. The findings of this study also confirm those reported by Alahamdi et al., where most Saudi females of reproductive age who had completed secondary school reported post-COVID-19 vaccination abnormalities in their menstrual cycle [30].

Approximately 989 (42%) study participants reported clot formation, and 745 (31%) reported heavy menstrual flow following COVID-19 vaccination. These findings align with a study that reported 35.3% of study participants experiencing heavy blood flow [15]. However, another retrospective study contradicted these findings and showed no significant changes in menstrual flow [20]. Additionally, there was a significant increase in pain intensity, with 1,127 (50%) participants experiencing pain after COVID-19 vaccination compared to 1,038 (46%) participants before vaccination.

## Conclusions

There is a high frequency of menstrual irregularities among females after receiving the COVID-19 vaccination. The most frequent abnormality is the irregularity in the length of the cycle after receiving two doses of COVID-19 vaccines. To draw more conclusive results, prospective studies with larger sample sizes are needed to investigate the long-term effects of COVID-19 vaccination on the reproductive health of females, especially in light of the emerging phenomenon of "long haul COVID-19," where individuals have been reported to visit hospitals with various infections even after recovering from COVID-19 and testing negative.
